# Integrated Analysis of Cell-Free DNA and Novel Protein Biomarkers for Stratification and Therapy Monitoring in Stage IV Pancreatic Cancer: A Preliminary Study

**DOI:** 10.3390/diagnostics15010049

**Published:** 2024-12-28

**Authors:** Saskia Hussung, Maria E. Hess, Elham Bavafaye Haghighi, Uwe A. Wittel, Melanie Boerries, Ralph M. Fritsch

**Affiliations:** 1 Department of Medicine I (Hematology, Oncology and Stem Cell Transplantation), Freiburg University Medical Center, 79106 Freiburg, Germany; saskia.hussung@usz.ch; 2 Department of Medical Oncology and Hematology, Zurich University Hospital, 8091 Zurich, Switzerland; 3 Institute of Medical Bioinformatics and Systems Medicine, Medical Center—University of Freiburg, Faculty of Medicine, University of Freiburg, 79104 Freiburg, Germany; maria.elena.hess@uniklinik-freiburg.de (M.E.H.); elham.bavafaye@mpinat.mpg.de (E.B.H.); melanie.boerries@uniklinik-freiburg.de (M.B.); 4 Faculty of Biology, University of Freiburg, 79104 Freiburg, Germany; 5 Department of Surgery, Freiburg University Medical Center, 79106 Freiburg, Germany; uwe.wittel@uniklinik-freiburg.de; 6 German Cancer Research Center (DKFZ), 69120 Heidelberg, Germany; 7 German Cancer Consortium (DKTK), Partner Site Freiburg, German Cancer Research Center (DKFZ), 69120 Heidelberg, Germany; 8 Comprehensive Cancer Center Freiburg (CCCF), Medical Center—University of Freiburg, Faculty of Medicine, University of Freiburg, 79104 Freiburg, Germany

**Keywords:** liquid biopsy, circulating tumor DNA, cell-free DNA (cfDNA), metastatic pancreatic adenocarcinoma (mPDAC), digital droplet PCR (ddPCR), protein biomarkers, *KRAS*, precision medicine

## Abstract

**Background:** Given the poor prognosis of metastatic pancreatic adenocarcinoma (mPDAC), closer disease monitoring through liquid biopsy, most frequently based on serial measurements of cell-free mutated *KRAS* (*KRAS*^mut^ cfDNA), has become a highly active research focus, aimed at improving patients’ long-term outcomes. However, most of the available data show only a limited predictive and prognostic value of single-parameter-based methods. We hypothesized that a combined longitudinal analysis of *KRAS*^mut^ cfDNA and novel protein biomarkers could improve risk stratification and molecular monitoring of patients with mPDAC. **Methods:** We prospectively collected 160 plasma samples from 47 patients with mPDAC at our institution. Highly sensitive single-target ddPCR assays were employed to detect and quantify *KRAS*^mut^ cfDNA. Additionally, analysis of ten protein biomarkers was performed through Enzyme-linked Immunosorbent Assay (ELISA), and Carbohydrate-Antigen 19-9 (CA 19-9) dynamics were registered. **Results**: *KRAS*^mut^ cfDNA was detectable in 37/47 (78.7%) patients throughout the course of study, and CA 19-9 levels were elevated in 40 out of 47 (85.1%) patients. *KRAS*^mut^ cfDNA increase at the time of the first follow-up could predict inferior progression-free survival (PFS) (Hazard ratio (HR) = 3.40, *p* = 0.0003) and overall survival (OS) (HR = 4.91, *p* < 0.0001). In contrast to CA 19-9 kinetics, which were not predictive of outcome, integrated analysis of *KRAS*^mut^ cfDNA combined with six evaluated circulating protein biomarkers allowed basal risk stratification at the time of the first follow-up (HR = 10.2, *p* = 0.0014). **Conclusions**: A combined longitudinal analysis of *KRAS*^mut^ cfDNA with selected protein biomarkers offers significantly improved prognostic value for patients with mPDAC compared to single-parameter methods. This innovative approach is a step forward in the molecular monitoring of mPDAC and should be validated in further prospective studies.

## 1. Introduction

Clinical management of patients diagnosed with pancreatic adenocarcinoma (PDAC) remains very challenging due to limited therapeutic options and a lack of biomarkers to better guide systemic treatment and predict the clinical course of disease. Even today, most patients present with stage IV disease and succumb to it within one year of diagnosis [1,2]. Although the disease is perceived as uniformly aggressive, there is great heterogeneity among patients in terms of response to treatment. While genomic studies have shown that pancreatic cancer is predominantly characterized by mutations in the four genes *KRAS*, *CDKN2A*, *TP53,* and *SMAD4*, it remains unclear how clinical heterogeneity arises when most tumors are caused by the same mutation pathway [3,4,5]. The lack of predictive and prognostic biomarkers, and the impossibility of detecting intrinsic resistance of the disease to molecularly targeted therapy, makes it difficult to develop personalized treatment strategies.

Even though classical protein-based tumor markers (CA 19-9, CEA) have been used for years to monitor the clinical course of disease in patients with known cancers, they are not suitable for screening purposes due to their sometimes low sensitivity or specificity [6,7,8,9,10]. A promising novel tool that has become the focus of the biomarker field in recent years is the detection of circulating nucleic acid fragments, particularly circulating tumor DNA (ctDNA), in the bloodstream of cancer patients [11,12,13,14]. This non-invasive blood-based technology can not only reflect tumor changes under therapy in real time [15] but also allows statements to be made regarding therapy response through longitudinal monitoring during the course of disease [15,16,17,18,19,20,21,22,23,24]. Highly sensitive detection methods, such as digital droplet PCR, allow sensitivities of 0.01 to 0.001% [25,26].

An alternative concept is the establishment of protein expression patterns from serum or plasma that may have the potential to map tumor-specific signatures from a few milliliters of blood [27,28]. A general observation, however, is that each individual method—whether protein-, DNA-, or RNA-based—has its specific advantages and limitations, and possibly only the combination and integration of different methodological approaches can meet all clinical requirements. The biomarker field is thus moving toward a combination of complementary approaches [27,29]. Therefore, the aim of this exploratory biomarker study was to investigate the potential of integrated biomarker analysis for clinical application in PDAC through the combined highly sensitive detection of cell-free mutated *KRAS* (*KRAS*^mut^ cfDNA) and a protein biomarker signature that we have already been able to establish in a systems biology approach [30].

## 2. Materials and Methods

### 2.1. Human Samples and Patient Cohort

Blood samples were collected from patients with clinically and histologically confirmed metastatic PDAC (mPDAC) at the time of diagnosis and during routine clinical follow-up, in addition to clinico-pathologic, treatment and outcome data. The diagnosis of metastatic disease was based on radiological and cyto-/histological confirmation. A total of 47 patients undergoing first-line systemic treatment or best supportive care following the diagnosis of mPDAC were recruited at the University Medical Center in Freiburg. All patients gave written informed consent to the collection and analysis of blood samples. The local institutional review board (IRB) approved all relevant procedures and analyses (EK-Freiburg project number 46/18). Treatment was performed as per standard of care and was blinded to KRASmut cfDNA results. Follow-up included 3-monthly clinical and radiological examinations using computed tomography and/or magnetic resonance imaging. Progressive disease (PD) was determined based on routine evaluation of radiological imaging.

### 2.2. Extraction of Cell-Free DNA from Plasma Samples

Venous blood samples from mPDAC patients were collected using commercially available EDTA tubes. Plasma was extracted through two subsequent centrifugation steps at 3000 rpm and 14,000 rpm for 10 min at 4 °C within one hour of collection, as previously described [31], and frozen at −80 °C without further treatment until extraction of cell-free DNA. CfDNA was extracted from each 4 mL plasma sample following the SEP/SBS protocol of the PME-free circulating DNA extraction kit (Analytik Jena, Jena, Germany, cat. no. 845-IR-0003050) according to the manufacturer’s instructions. Two subsequent elution steps with 30 µL Elution Buffer were performed. DNA was stored at −20 °C until ctDNA quantification. CfDNA was evaluated with a fragment analyzer and quantified using the Qubit 2.0 fluorometer. DNA yield from 4 mL of plasma typically ranged from 1–20 ng/µL to up to 80 ng/µL for metastatic pancreatic cancer patients.

### 2.3. Enzyme-Linked Immunosorbent Assay (ELISA)

ELISA antibodies and preparation kits for SFN and TFF1 were manufactured by Wuhan USCN Business (USCN, Co., Ltd., Wuhan, China; cat. no. #SEH179Hu, #SEB049Hu) and for CEMIP, COL10A1, HGF, LAMB3, POSTN, SERPINB5, SPP1, and TMPRSS4 by LifeSpan Biosciences (LSBio, Seattle, WA, USA; cat. no. #LS-F7390, # LS-F13131, #LS-F2441, #LS-F11919, #LS-F3645, #LS-F13455, #LS-F13367, #LS-F7623). Plasma was frozen at −80 °C before preparation according to the manufacturers’ instructions. The colorimetric reactions were analyzed on a Tecan Infinite M200 Pro.

### 2.4. Droplet Digital PCR (ddPCR)

Locked nucleic acid (LNA)-based probes and associated primer pairs for detecting *KRAS* mutations in plasma cfDNA were developed using Beacon Designer v.8.20 software (Premier Biosoft, Palo Alto, CA, USA). These primers and probes were custom-synthesized by Integrated DNA Technologies (IDT, Inc., Coralville, IA, USA). Comprehensive details regarding the design process, including sequences, have been previously published [31]. The reaction mixture for droplet digital PCR (ddPCR) was prepared by combining ddPCR Supermix for Probes (Bio-Rad, Hercules, CA, USA, catalog #186-3024) with primers, probes, 2 µL of template DNA per well, and nuclease-free water (Ambion, Austin, TX, USA). Droplets were created using the QX100/200™ Droplet Generator (Bio-Rad, catalog #1863002) following the manufacturer’s protocol. Each sample was analyzed in four replicates. After generating the droplets, the mixture was transferred to a 96-well PCR plate (Bio-Rad, catalog #12001925) and PCR was run on a C1000 Touch™ Thermal Cycler (Bio-Rad, catalog #1851197). The amplified droplets were subsequently analyzed using the QX100/200™ Droplet Reader (Bio-Rad, catalog #1863003) with the QuantaSoft software v1.7.4.0917 (Bio-Rad, catalog #1864011). Details of the PCR protocols, assay controls, and data analysis methods for the *KRAS* assays have been previously published [31]. The number of copies per milliliter of plasma was determined using the following calculation [31]:

$$\mathrm{copies}/\mathrm{mL}\;\mathrm{plasma}=-\;\ln\;(\frac{N_{neg}}{N})/\;V_{droplet}\;\times \;20\;µL\;\times \;7.5\;$$

### 2.5. Statistical Analysis

The primary outcome measured was Progression-free survival (PFS), based on routine evaluation of radiological imaging. PFS was defined from the start of first-line therapy to the verified first radiologic progression, based on standard restaging imaging or death due to any cause. For this purpose, patients were divided into two groups based on their radiological response: patients with complete or partial remission and patients with stable disease were assigned to the “non-PD” (non-Progressive Disease) group and compared to patients with “PD”. Overall Survival (OS) was the secondary endpoint and was defined as the time from the date of diagnosis to death. Kaplan–Meier survival analysis was performed to estimate progression-free and survival time. Univariate analyses were carried out using the log-rank test. Backward stepwise Cox regression modeling to estimate the hazard ratio (HR) with a 95% confidence interval (CI) was used to explore independent prognostic factors for PFS and OS. Fisher’s exact test and the Wilcoxon–Mann–Whitney test were carried out to compare independent variables. All statistical analyses were performed using GraphPad Prism Version 9.4.0 (GraphPad Software, Inc., La Jolla, CA, USA) and SPSS 26 software Version 26.0.0.0 (IBM Corporation, North Castle, NY, USA). *p* values < 0.05 were considered significant.

### 2.6. Risk Stratification and Classification

The protein levels were determined for CEMIP, COL10A1, HGF, LAMB3, POSTN, SERPINB5, SFN, SPP1, TFF1, and TMPRSS4. In order to illustrate the concentration level of each marker, the level of the markers were scaled independently. In the case of COL10A1, TMPRSS4, CEMIP, and *KRAS*^mut^ cfDNA, six levels with drastically higher values in comparison to the other outliers were excluded to make the general trend of the biomarkers observable. To consider the effect of therapy in respect to OS and the kinetics of protein levels, a binary variable was provided for each protein as follows: The binary variable represents whether the level of that protein increases or decreases after therapy starts for the corresponding patient. For increasing protein levels “1” is used, and “−1” for decreasing one. For patients with more than one follow-up sample, we decided for the increasing or decreasing trend that most of the samples showed. In the case of ties, the first follow-up’s binary value was used. We performed the same procedure on the *KRAS*^mut^ cfDNA levels. Each univariate model stratified the patients based on the binary values independently and was evaluated using the log-rank test.

In order to identify a unified signature of the markers using a multivariate CoxPH model [32], the R package GLMnet was used with LASSO and 3-fold cross-validation based on the combination of the binary variables [33,34]. Risk labels were computed using the estimated model. The prognostic potential of the gained model was evaluated using the log-rank test.

Using the labels of low and high-risk for the patients in the PT-and-UT group provided by the multivariate CoxPH model, a Risk Classifier was trained to classify patients into the corresponding risk groups. The learning process used the protein levels of the selected biomarkers of the prior to treatment condition as the feature space and the risk labels of the PT-and-UT patients as the labels. Binomial Logistic Regression [33] was applied for learning the classifier with alpha = 0.6 for the setting of the elastic net and 4-fold cross-validation. Higher absolute values of the coefficients increase the impact of the corresponding variable on the Risk Classifier.

The Risk Classifier then predicted the risk label of each of the PT-and-UT and PT-only patients. Three patients in the PT-and-UT group were misclassified (error rate 16%). In the case of PT-only group, the predicted risk labels were used to provide a Kaplan–Meier plot, where the stratification significance was evaluated using log-rank tests.

## 3. Results

### 3.1. Patient Characteristics

Forty-seven patients with clinically and histologically confirmed metastatic PDAC (mPDAC) were included in the study. Clinico-pathological parameters and basic clinical characteristics of these stage IV PDAC patients are summarized in Supplemental Tables S1 and S2. 42/47 patients (89.4%) received first-line chemotherapy, and 5/47 (10.6%) underwent best supportive care (BSC). The median follow-up time among surviving patients was 9.5 months (95% CI 3.0–20.0 months). The median progression-free survival (PFS) was 3.0 months (95% CI 2.0–6.0 months), and the median overall survival (OS) was 9.0 months (5.0–13.0 months). At the time of the final analysis, 41/47 of the patients (87.2%) had died.

### 3.2. Analysis of Plasma KRAS^mut^ cfDNA

A total of 160 blood samples from 47 patients with mPDAC were analyzed in this study using ddPCR. The first samples were taken at a median of 7.5 days (95% CI 4–11) prior to the start of first-line treatment. The median number of samples collected was three samples per patient (95% CI 2–4). The median time interval between the start of therapy and the first follow-up was 55 days (95% CI 50–62). The mutant *KRAS* detection rate at baseline was 70.21% (33/47). A correlation between *KRAS*^mut^ cfDNA positivity and the presence of liver metastases was shown (*p* = 0.0015, Supplemental Table S3). PDAC patients with liver metastases also presented significantly higher *KRAS*^mut^ cfDNA copies/mL plasma than patients with metastases located elsewhere (*p* = 0.0003; Supplemental Figure S1A). For the tumor marker CA 19-9, this difference was less pronounced (*p* = 0.0178; Supplemental Figure S1C). Regarding tumor grading, there was no difference between well and poorly differentiated tumors and the amount of *KRAS*^mut^ cfDNA or CA 19-9 levels (Supplemental Figure S1B,D). Although *KRAS*^mut^ cfDNA positive patients had higher levels of the tumor marker CA 19-9 (*p* = 0.0054; Supplemental Figure S1E), there was generally no correlation between the CA 19-9 level and the amount of *KRAS*^mut^ cfDNA in plasma (Supplemental Figure S1F).

### 3.3. Univariate and Multivariate Analyses of PFS and OS in mPDAC Patients

For the univariate analysis of OS in our cohort of mPDAC patients, 11 independent demographic and clinico-pathologic features were examined, which are presented in Table 1. Four independent variables—namely, tumor differentiation, the number of metastatic sites, administration of systemic treatment, and change in levels of *KRAS*^mut^ cfDNA during chemotherapy—were identified as prognostic factors. The multivariate Cox proportional hazards regression model showed that the *KRAS*^mut^ cfDNA increase at time of the first follow-up (Hazard ratio (HR) = 10.9, 95% CI: 2.589–46.17, *p* = 0.001) and tumor differentiation (HR = 3.17, 95% CI: 1.175–8.535, *p* = 0.023) were the only significant factors for survival in our cohort of mPDAC patients (Table 1). Corresponding analyses were also performed for PFS and are presented in Supplemental Table S4. Again, the multivariate Cox proportional hazards regression model showed that *KRAS*^mut^ cfDNA increase at the time of the first follow-up (HR = 10.9, 95% CI: 2.575–46.444, *p* = 0.001) was a significant factor for PFS. Furthermore, the number of metastatic sites (HR = 7.20, 95% CI: 1.149–45.080, *p* = 0.035), older age (HR = 4.39, 95% CI: 1.250–15.42, *p* = 0.021), tumor location (HR = 0.20, 95% CI: 0.053–0.721, *p* = 0.014), and CA 19-9 positivity in plasma before the start of systemic treatment (HR = 4.38, 95% CI: 1.180–16.285, *p* = 0.027) were also shown to be significant variables for PFS.

### 3.4. Predictive and Prognostic Value of KRAS^mut^ cfDNA and CA 19-9

To evaluate the predictive and prognostic relevance of *KRAS*^mut^ cfDNA and CA 19-9 levels, baseline values of these biomarkers before the start of palliative first-line treatment were correlated with PFS and OS. High *KRAS*^mut^ cfDNA levels (>25 copies/mL) before the start of systemic chemotherapy were inversely associated with PFS (HR = 2.07, 95% CI: 1.011–4.229, *p* = 0.0057; Supplemental Figure S2A) and also showed a trend toward inferior OS (HR = 1.72, 95% CI: 0.850–3.489, *p* = 0.0775; Supplemental Figure S2B). In addition, detection of a high amount of total cfDNA in plasma was associated with shorter PFS in our study cohort (HR 2.56, 95% CI: 1.217–5.367, *p* = 0.0002; *p* < 0.0001; Supplemental Figure S3A), but had no prognostic value (Supplemental Figure S3B). ctDNA positivity before palliative first-line chemotherapy was not associated with inferior PFS and OS in our study cohort (Supplemental Figure S3C,D). Analysis of the tumor marker CA 19-9 prior to therapy also had no predictive or prognostic value in our cohort (Supplemental Figure S2C,D). A threshold value of 100 U/mL was chosen because previous data showed that this value is not found in healthy individuals without a clinical history of cancer disease [27,35].

### 3.5. Association of KRAS^mut^ cfDNA and CA 19-9 Dynamics with Survival

**Figure 1:**
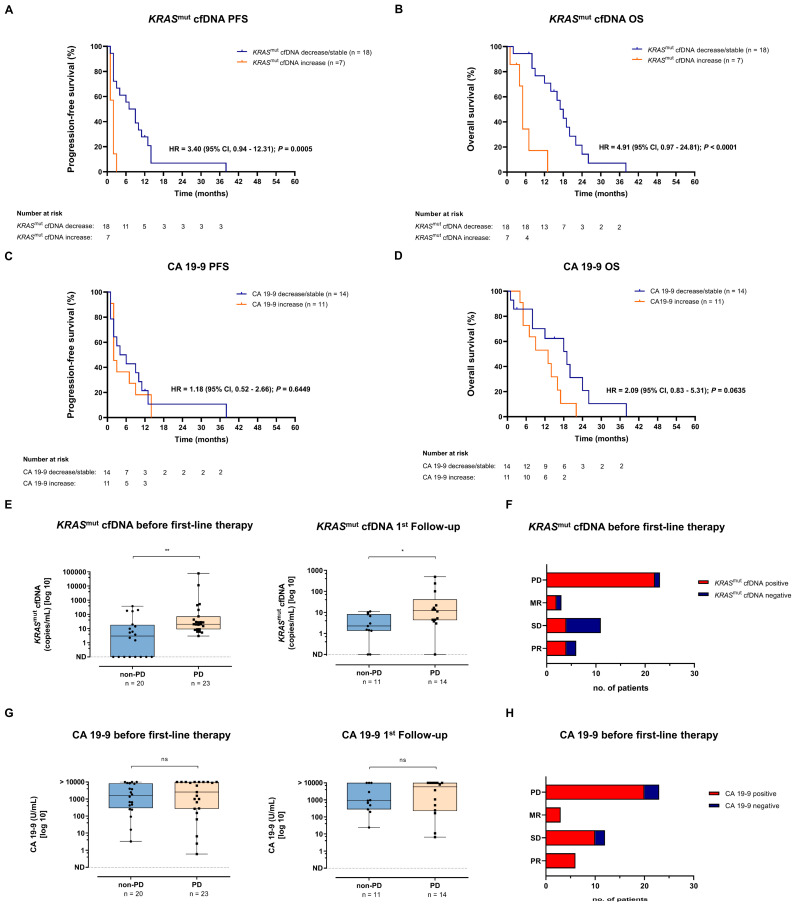
Progression-free survival (PFS) and overall survival (OS) analyses for metastasized PDAC patients undergoing first-line systemic treatment. (**A**,**B**) Kaplan–Meier estimates of PFS (**A**) and OS (**B**) for metastasized PDAC patients stratified by change in *KRAS*^mut^ cfDNA levels at the time of first restaging: *KRAS*^mut^ cfDNA decrease/stable versus increase. (**C**,**D**) Kaplan–Meier estimates of PFS (**C**) and OS (**D**) for metastatic PDAC patients stratified by change in CA 19-9 levels at the time of first follow-up: CA 19-9 decrease/stable versus increase. (**E**) Left: Association of *KRAS*^mut^ cfDNA levels prior to the start of systemic treatment with radiologic response at first restaging (non-progressive vs. progressive disease). Right: Association of *KRAS*^mut^ cfDNA levels and radiologic response to first-line therapy at first follow-up. (**F**) Association of *KRAS*^mut^ cfDNA positivity prior to the start of systemic treatment with radiologic response at first restaging (non-progressive vs. progressive disease). (**G**) Left: Association of CA 19-9 levels prior to start of systemic treatment with radiologic response at first restaging (non-progressive vs. progressive disease). Right: Association of CA 19-9 levels and radiologic response to first-line therapy at first follow-up. (**H**) Association of CA 19-9 positivity prior to start of systemic treatment with radiologic response at first restaging (non-progressive vs. progressive disease). MR, mixed response; OS, overall survival; PD, progressive disease; PDAC, pancreatic adenocarcinoma; PFS, progression-free survival; PR, partial response; SD, stable disease; ns, not significant. * *p* < 0.05, ** *p* < 0.01.

Protein tumor markers and *KRAS*^mut^ cfDNA are highly dynamic biomarkers. Therefore, in the next step, a second blood sample was analyzed at the time of the first follow-up (median after 55 days) and biomarker changes during this time interval were correlated with PFS and OS. For 25/47 (53.2%) patients, a sufficient number of follow-up samples was available for subsequent analysis. The increase of *KRAS*^mut^ cfDNA was associated with significantly reduced PFS and OS (Figure 1A,B), while the increase of the total amount of cell-free DNA did not correlate with PFS or OS (Supplemental Figure S3E,F). The increase of the tumor marker CA 19-9 was associated with a non-significant trend toward inferior OS but not PFS (Figure 1C,D). Furthermore, the radiological imaging-based division into progressive disease (PD) and non-PD at the time of first restaging did not show any difference regarding OS (Supplemental Figure S2G). Interestingly, patients with PD at the time of the first follow-up already showed elevated *KRAS*^mut^ cfDNA levels in plasma prior to first-line therapy (Figure 1E, left). Also, at the time of the first follow-up, they showed significantly higher *KRAS*^mut^ cfDNA levels in plasma (Figure 1E right). On the contrary, no difference was found for the tumor marker CA 19-9 (Figure 1G). Corresponding to this, 22/23 patients (95.65%) who presented with PD at the time of the first follow-up were positive for *KRAS*^mut^ cfDNA before the start of first-line therapy, whereas 7/11 patients (63.64%) with stable disease were *KRAS*^mut^ cfDNA negative (Figure 1F). For the tumor marker CA 19-9, no correlation was found (Figure 1H).

### 3.6. Clinical Response Prediction by Kinetics of KRAS^mut^ cfDNA and CA 19-9

**Figure 2:**
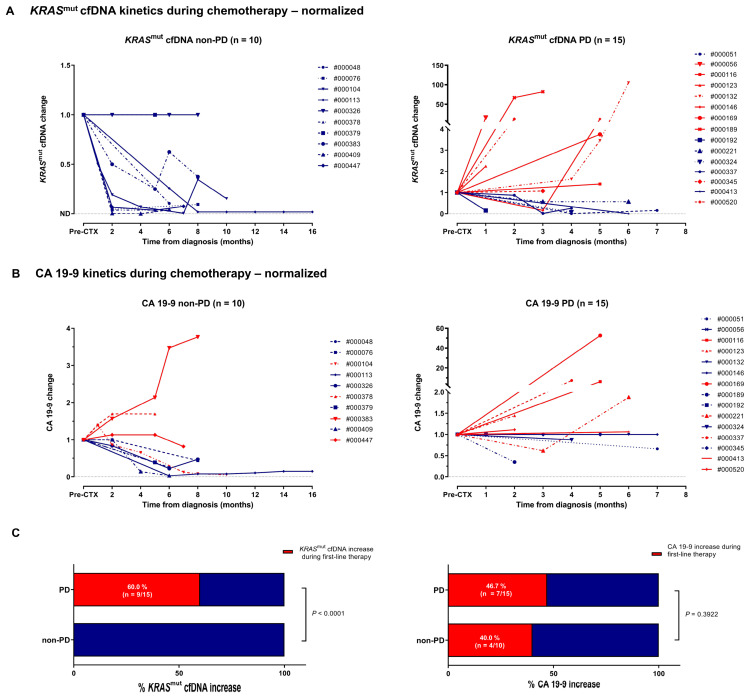
Clinical response prediction by kinetics of *KRAS*^mut^ cfDNA and CA 19-9. (**A**,**B**) According to their radiological response to first-line therapy, metastatic PDAC patients were divided into two groups: patients with non-PD vs. with PD. Absolute levels of *KRAS*^mut^ cfDNA (**A**) and CA 19-9 (**B**) were normalized to the respective pre-treatment levels for each individual patient. (**C**) PD vs. non-PD patients with an increase in *KRAS*^mut^ cfDNA or CA 19-9 level during the observation period. Fisher’s exact test was used to interrogate statistical significance between the two groups. *p* values < 0.05 were considered significant.

**Figure 3:**
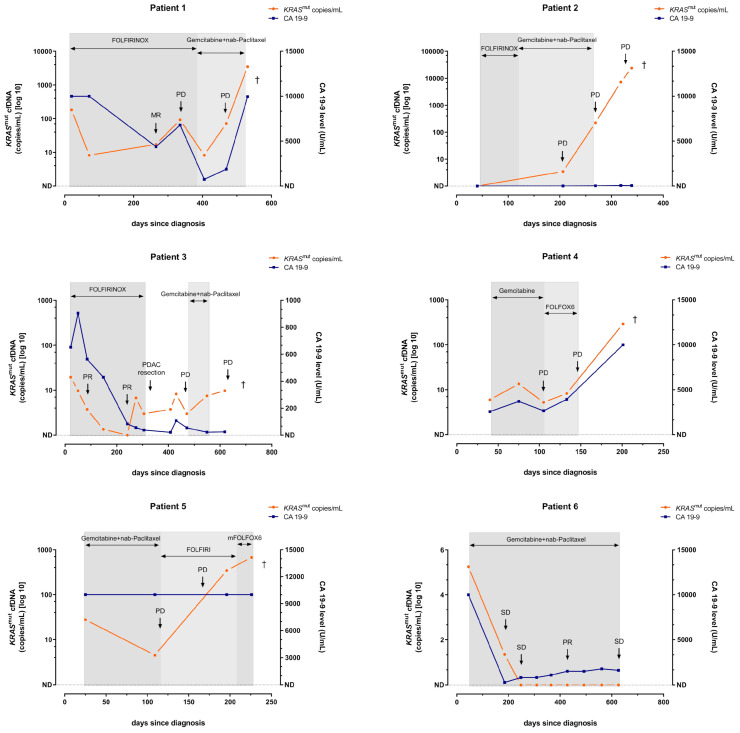
Single-patient analysis of *KRAS*^mut^ cfDNA and CA 19-9 dynamics. *KRAS*^mut^ cfDNA and CA 19-9 levels during palliative chemotherapy for metastatic PDAC patients. Radiological staging was performed every 3 months using computed tomography or magnetic resonance imaging. Shades of gray in the background reflect the changes in treatment conditions. † patient death.

Figure 2A,B illustrates the correlation between *KRAS*^mut^ cfDNA and CA 19-9 dynamics during first-line therapy and radiologic response to treatment at the time of the first follow-up. 13/25 patients (52%) showed PD at the first restaging. The increase of *KRAS*^mut^ cfDNA was significantly associated with PD and outperformed CA 19-9 as dynamic marker (Figure 2C). Overall, the kinetics of *KRAS*^mut^ cfDNA analyses nicely reflected individual patients’ course of disease, as illustrated in 6/6 exemplary shown cases (Figure 3). Individual patient analyses showed that *KRAS*^mut^ cfDNA is a highly dynamic biomarker, which can be clinically especially relevant for patients with non-elevated CA 19-9 levels. However, prospective studies and larger cohorts are needed to better unravel the temporal relationship between biomarker dynamics and the clinical disease course and to investigate the impact on therapeutic strategy.

### 3.7. Risk Stratification Based on the Kinetics of KRAS^mut^ cfDNA and Biomarker Proteins

**Figure 4:**
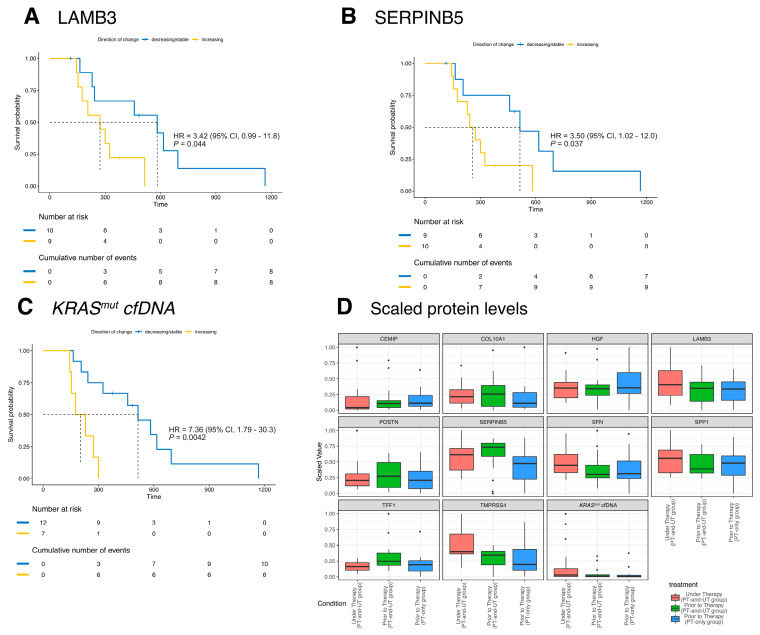
Survival analysis for concentration changes for LAMB3, SERPINB5, and *KRAS*^mut^ cfDNA for the PT-and-UT group. (**A**–**C**) Kaplan–Meier plot for patients of the PT-and-UT group for LAMB3 (**A**), SERPINB5 (**B**), and *KRAS*^mut^ cfDNA (**C**). Groups were divided by proteins’ development under therapy: decreasing/stable (blue), increasing (yellow). The log-rank test was used to provide the statistical significance of the stratification of the two groups. (**D**) Protein levels were measured via Enzyme-linked Immunosorbent Assay (ELISA) for two subgroups of patients. In order to illustrate the concentration level of each of the markers, the levels of the markers were scaled independently.

Out of the total 47 patients in this study, only 19 cases had enough plasma left for ELISA measurements prior to and under therapy (PT-and-UT group). For a set of 17 patients plasma samples prior to therapy were available (PT-only group) and therefore used as the validation set. In order to improve the prognostic impact of *KRAS*^mut^ cfDNA in mPDAC, the role of ten proteins in plasma (CEMIP, COL10A1, HGF, LAMB3, POSTN, SERPINB5, SFN, SPP1, TFF1, and TMPRSS4) was investigated by considering the effect of the first-line therapy. The related genes were previously described as a subset of relevant biomarkers in distinguishing PDAC tissue from normal tissue by RNA sequencing and showed significantly elevated levels in plasma samples of PDAC patients [30]. To examine the prognostic role of these proteins in mPDAC, we referred to the protein levels measured via Enzyme-linked Immunosorbent Assay (ELISA) of two subgroups of patients (scaled values in Figure 4D).

As a first step of our analysis, we examined the single biomarker impact on the OS of the prior to and under treatment mPDAC patients (PT-and-UT group) using the univariate HR model. Most of the single biomarkers were not able to stratify patients significantly (Supplemental Table S5). However, the prognostic impact of the single proteins LAMB3 and SERPINB5 was significant, although the corresponding significance was not high (*p* = 0.044; *p* = 0.037, labeled with a star, Supplemental Table S5, Figure S4A,B). The prognostic impact of *KRAS*^mut^ cfDNA was the highest among all of the evaluated markers (*p* = 0.0042, Figure 4C).

**Figure 5:**
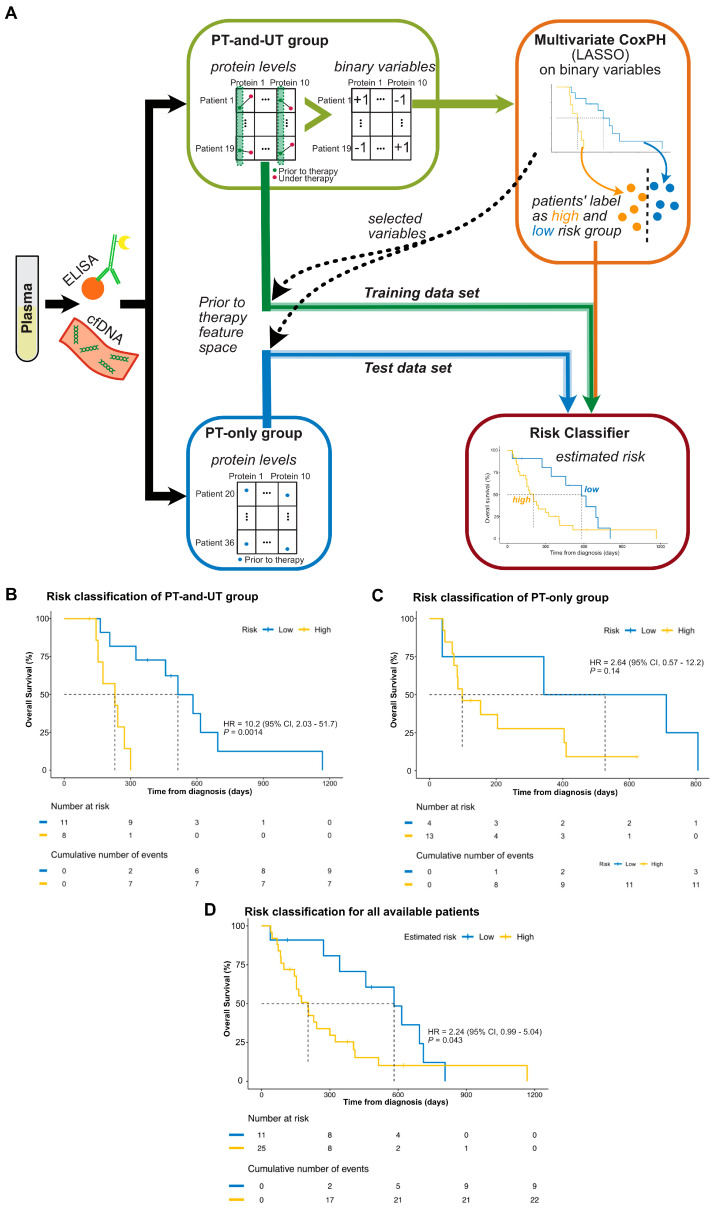
Risk stratification based on the kinetics of biomarkers. (**A**) Scheme of the risk stratification. Patients’ plasma samples were used for the determination of cfDNA and biomarker protein levels. Samples were divided into PT-and-UT and PT-only groups. Binarized PT-and-UT group values were used for the multivariate CoxPH model regularized using LASSO to identify biomarkers and categorize patients into high- and low-risk groups. The provided labels by CoxPH, together with the prior to therapy protein levels, were further used to train the Risk Classifier. Protein levels of the PT-only group were used as the test dataset for the Risk Classifier. The feature selection of the test and training data was accomplished based on the biomarkers identified by the CoxPH (LASSO). (**B**) By applying the multivariate CoxPH model, and based on the impact of the therapy on the kinetics of six proteins and *KRAS*^mut^ cfDNA, PT-and-UT group patients were stratified into low (blue) and high risk (yellow) groups. This figure presents the details of the brown box of Figure 4A. (**C**) Kaplan–Meier plot of the PT-only group, in which the risk label of the patients was estimated using the Risk Classifier. (**D**) Kaplan–Meier plot of all available patients, including training and test sets, in which the related risk labels were estimated by the Risk Classifier. The log-rank test was used to provide the statistical significance of the stratification of the two groups in the plots of (**B**–**D**).

Based on the above results, we used a multivariate Cox Proportional Hazard (CoxPH) model to identify the prognostic biomarkers together with *KRAS*^mut^ cfDNA based on the PT-and-UT group [32,33]. Similar to the previous univariate models, we used the binary variables to consider the kinetics of each protein and the effect of therapy. However, the binary variables were used together to identify a unified signature of the biomarker proteins and *KRAS*^mut^ cfDNA (Figure 5A). Figure 5B shows the result of the multivariate CoxPH to stratify patients into high- or low-risk groups (HR = 10.2, 95% CI: 2.03–51.7, *p* = 0.0014). The significance of the multivariate CoxPH model was improved in comparison to each of the univariate ones. The selected set of prognostic biomarkers identified using LASSO and their related impacts are given in Supplemental Table S6. In detail, the selected set of biomarkers by CoxPH includes *KRAS*^mut^ cfDNA and six other proteins: CEMIP, TFF1, LAMB3, HGF, TMPRSS4, and SERPINB5. CEMIP was the only significant biomarker, and its impact was higher than that of *KRAS*^mut^ cfDNA.

To predict the therapy response in respect to OS, we used the selected set of markers to classify eligible patients. Patients predicted to be in the low-risk group are more likely to benefit from further therapy. Based on the selected markers and using the prior to therapy samples of the PT-and-UT group, together with the identified label of low or high risk given by the multivariate CoxPH, a Risk Classifier was provided to predict the label of risk (Supplemental Table S7). The accuracy of the Risk Classifier for the PT-and-UT group is 84%. The actual risk label of each PT-and-UT patient and the predicted one by the Risk Classifier are given in Supplemental Table S8.

The Risk Classifier was applied to recognize the risk label of the PT-only group to predict the therapy response in respect to OS. The OS Kaplan–Meier plot of Figure 5C, based on the predicted risk labels by the Risk Classifier, presents an acceptable separation within the PT-only group, although not significant (*p* = 0.17). We believe that a higher number of patients, especially with under-therapy samples, are necessary to validate the Risk Classifier. However, the Risk Classifier showed a significant prognostic performance for all available patients’ data of prior to therapy samples (*p* = 0.043, Figure 5D).

## 4. Discussion

In this single-center exploratory study, we followed a cohort of 47 mPDAC patients undergoing palliative first-line treatment or BSC at our institution. We analyzed *KRAS*^mut^ cfDNA using previously established, highly sensitive single-target and discriminatory multi-target ddPCR assays [31]. Results were integrated with CA 19-9 levels and ten protein biomarkers (CEMIP, TFF1, LAMB3, HGF, TMPRSS4, SERPINB5, SPP1, POSTN, TFF1, COL10A1) for association with progression-free and overall survival endpoints. Over the past decades numerous studies have demonstrated the predictive and prognostic significance of ctDNA for closer disease monitoring in pancreatic cancer [19,20,21,23,24,36,37,38,39,40]. To our best knowledge, even today, there are only limited data on the serial analysis of ctDNA in advanced or mPDAC available [20,21,22,24]. What sets our study apart is the combined longitudinal analysis of *KRAS*^mut^ cfDNA and novel protein biomarkers, overcoming the limitations of single-parameter-based methods, enabling risk stratification, and molecular monitoring of patients with mPDAC. Our study highlights the importance of *KRAS*^mut^ cfDNA serial monitoring as a sensitive and highly specific biomarker for therapy response and disease progression. The use of discriminatory multi-target ddPCR assays hereby allows for direct KRAS SNV detection without prior tumor NGS, making it possible to assess tumor dynamics in a time-relevant co-clinical setting analogous to CA 19-9 levels [31,41]. An increase in *KRAS*^mut^ cfDNA during first-line palliative treatment in our study cohort was significantly associated with shorter progression-free and overall survival and outperformed CA 19-9 kinetics and standard imaging. In particular, for patients not expressing CA 19-9 or lacking dynamic changes of the tumor marker during systemic treatment, the analysis of ctDNA in plasma might provide additional diagnostic benefit. However, each individual method has its specific advantages and limitations, and possibly only the combination and integration of different methodological approaches can meet all clinical requirements. For our cohort the integrated analysis of *KRAS*^mut^ cfDNA combined with six evaluated circulating protein biomarkers allowed basal risk stratification at the time of first follow-up. Yet, routine clinical assessment of dynamic ctDNA changes and protein expression patterns during systemic treatment of mPDAC using a standardized approach is lacking.

Still today, systemic treatment options for pancreatic cancer are limited to a small number of combination chemotherapy regimens [42,43] and new attempts at personalized treatment based on molecular profiling [44,45]. Given the fact that, according to the current literature, only approximately 50% of PDAC patients receive further line treatment, an early switch of chemotherapy in case of lack of response is crucial [42,46]. Thus, we advocate serial ctDNA analysis to assess response dynamics within a time-relevant framework. *KRAS*^mut^ cfDNA-guided therapy monitoring in pancreatic cancer appears feasible, but still requires extensive validation in prospective clinical trials to investigate whether treatment decisions based on serial ctDNA assessment result in improved outcomes for PDAC patients.

Nevertheless, there are also some limitations to our study. Subgroup analyses of the present study were performed with a relatively small number of metastasized PDAC patients, and survival outcomes were correlated retrospectively. Thus, the results are exploratory in nature and only hypothesis-generating. In contrast to previously published data [22,24,47,48,49], *KRAS*^mut^ cfDNA positivity prior to palliative first-line therapy was not associated with shorter PFS and OS in our cohort. However, high *KRAS*^mut^ cfDNA levels (>25 copies/mL) before the start of systemic chemotherapy were associated with significantly reduced PFS and showed a trend toward inferior OS, highlighting the importance of identifying clinically validated cut-offs for ctDNA analysis [18,50,51]. Interestingly, Del Re et al. [20] also found no association between *KRAS*^mut^ cfDNA positivity prior to palliative first-line therapy and survival endpoints. Also, in this study, highly sensitive ddPCR was used to detect ctDNA in plasma, and the *KRAS*^mut^ cfDNA detection rate of 70.4% at baseline was identical to the detection rate in our study, whereas in previously published studies, detectability ranged from 0% to 62.2% [48,49,52]. The significantly higher positivity rate could be another possible explanation for the lack of association with survival endpoints.

In summary, our pilot study proposed a clinically feasible way to assay *KRAS*^mut^ cfDNA in plasma together with novel protein biomarkers in mPDAC patients and outperformed CA 19-9. Through the combination of these markers, patients could be better stratified in terms of treatment response and overall prognosis.

## Figures and Tables

**Table 1:** Overall survival analysis by clinico-pathologic variables and KRAS^mut^ cfDNA status.

Variable	Univariate Analysis			Multivariate Analysis		
	HR	95% CI ^§^	*p*	HR	95% CI	*p*
Age ≥median vs. <median						
	1.27	0.686–2.334	0.4452			
Gender male vs. female						
	1.26	0.679–2.339	0.4318			
Tumor location pancreas body and tail vs. head						
	0.67	0.346–1.311	0.2122			
Tumor differentiation poor vs. well/medium						
	2.11	0.990–4.483	**0.0212**	3.17	1.175–8.535	**0.023**
Liver metastasis present vs. absent						
	1.67	0.889–3.139	0.1289			
No. of metastatic sites ≥2 vs. 1						
	1.86	0.866–4.005	**0.0499**			
Systemic treatment yes vs. no						
	0.32	0.071–1.462	**0.0111**			
CA 19-9 status >37 vs. ≤37 U/mL						
	0.60	0.211–1.725	0.2382			
*KRAS*^mut^ cfDNA status positive vs. negative						
	1.06	0.553–2.034	0.8533			
CA 19-9 during follow-up increase vs. decrease						
	2.23	0.827–6.032	0.0549			
*KRAS*^mut^ during follow-up increase vs. decrease						
	5.03	0.978–25.83	**<0.0001**	10.9	2.589–46.17	**0.001**

^§^ CI, confidence interval. All *p*-values < 0.05 are displayed in bold.

## Data Availability

The datasets used and/or analyzed during the current study are available from the corresponding author on reasonable request.

## Supplementary Materials

The following supporting information can be downloaded at: https://www.mdpi.com/article/10.3390/diagnostics15010049/s1, Figure S1: Plasma *KRAS*^mut^ cfDNA and CA 19-9 in metastasized PDAC patients; Figure S2: Association of *KRAS*^mut^ cfDNA and CA 19-9 detection with survival endpoints; Figure S3: Association of *KRAS*^mut^ cfDNA detection with survival endpoints; Table S1: Basic clinical characteristics of the study cohort; Table S2: Clinical characteristics of the individual patients; Table S3: *KRAS*^mut^ cfDNA status and radiological site of metastasis; Table S4: Progression-free survival analysis by clinico-pathologic variables and *KRAS*^mut^ cfDNA status; Table S5: Prognostic impact of the single proteins by considering the impact of therapy; Table S6: Significance of the selected biomarkers in the multivariate CoxPH model; Table S7: Significance of the selected biomarkers in the risk classifier; Table S8: Risk labels provided by CoxPH and by the risk classifier.

## References

[1] Ryan D.P., Hong T.S., Bardeesy N. (2014). Pancreatic Adenocarcinoma. N. Engl. J. Med..

[2] Rawla P., Sunkara T., Gaduputi V. (2019). Epidemiology of Pancreatic Cancer: Global Trends, Etiology and Risk Factors. World J. Oncol..

[3] Jones S., Zhang X., Parsons D.W., Lin J.C.-H., Leary R.J., Angenendt P., Mankoo P., Carter H., Kamiyama H., Jimeno A. (2008). Core signaling pathways in human pancreatic cancers revealed by global genomic analyses. Science.

[4] Witkiewicz A.K., McMillan E.A., Balaji U., Baek G., Lin W.-C., Mansour J., Mollaee M., Wagner K.-U., Koduru P., Yopp A. (2015). Whole-exome sequencing of pancreatic cancer defines genetic diversity and therapeutic targets. Nat. Commun..

[5] Waddell N., Pajic M., Patch A.-M., Chang D.K., Kassahn K.S., Bailey P., Johns A.L., Miller D., Nones K., Quek K. (2015). Whole genomes redefine the mutational landscape of pancreatic cancer. Nature.

[6] Locker G.Y., Hamilton S., Harris J., Jessup J.M., Kemeny N., Macdonald J.S., Somerfield M.R., Hayes D.F., Bast R.C. (2006). ASCO 2006 update of recommendations for the use of tumor markers in gastrointestinal cancer. J. Clin. Oncol. Off. J. Am. Soc. Clin. Oncol..

[7] Lennon A.M., Goggins M. (2010). Diagnostic and Therapeutic Response Markers. Pancreatic Cancer.

[8] Poruk K.E., Gay D.Z., Brown K., Mulvihill J.D., Boucher K.M., Scaife C.L., Firpo M.A., Mulvihill S.J. (2013). The clinical utility of CA 19-9 in pancreatic adenocarcinoma: Diagnostic and prognostic updates. Curr. Mol. Med..

[9] Ballehaninna U.K., Chamberlain R.S. (2012). The clinical utility of serum CA 19-9 in the diagnosis, prognosis and management of pancreatic adenocarcinoma: An evidence based appraisal. J. Gastrointest. Oncol..

[10] Goggins M. (2005). Molecular Markers of Early Pancreatic Cancer. J. Clin. Oncol..

[11] Alix-Panabières C., Pantel K. (2016). Clinical Applications of Circulating Tumor Cells and Circulating Tumor DNA as Liquid Biopsy. Cancer Discov..

[12] Bettegowda C., Sausen M., Leary R.J., Kinde I., Wang Y., Agrawal N., Bartlett B.R., Wang H., Luber B., Alani R.M. (2014). Detection of circulating tumor DNA in early- and late-stage human malignancies. Sci. Transl. Med..

[13] Diaz L.A., Bardelli A. (2014). Liquid biopsies: Genotyping circulating tumor DNA. J. Clin. Oncol. Off. J. Am. Soc. Clin. Oncol..

[14] Macías M., Alegre E., Díaz-Lagares A., Patiño A., Pérez-Gracia J.L., Sanmamed M., López-López R., Varo N., González A. (2018). Liquid Biopsy: From Basic Research to Clinical Practice. Adv. Clin. Chem..

[15] Diehl F., Schmidt K., Choti M.A., Romans K., Goodman S., Li M., Thornton K., Agrawal N., Sokoll L., Szabo S.A. (2008). Circulating mutant DNA to assess tumor dynamics. Nat. Med..

[16] Diaz L.A., Williams R.T., Wu J., Kinde I., Hecht J.R., Berlin J., Allen B., Bozic I., Reiter J.G., Nowak M.A. (2012). The molecular evolution of acquired resistance to targeted EGFR blockade in colorectal cancers. Nature.

[17] Misale S., Yaeger R., Hobor S., Scala E., Janakiraman M., Liska D., Valtorta E., Schiavo R., Buscarino M., Siravegna G. (2012). Emergence of KRAS mutations and acquired resistance to anti-EGFR therapy in colorectal cancer. Nature.

[18] Crowley E., Di Nicolantonio F., Loupakis F., Bardelli A. (2013). Liquid biopsy: Monitoring cancer-genetics in the blood. Nat. Rev. Clin. Oncol..

[19] Watanabe F., Suzuki K., Tamaki S., Abe I., Endo Y., Takayama Y., Ishikawa H., Kakizawa N., Saito M., Futsuhara K. (2019). Longitudinal monitoring of KRAS-mutated circulating tumor DNA enables the prediction of prognosis and therapeutic responses in patients with pancreatic cancer. PLoS ONE.

[20] Del Re M., Vivaldi C., Rofi E., Vasile E., Miccoli M., Caparello C., d’Arienzo P.D., Fornaro L., Falcone A., Danesi R. (2017). Early changes in plasma DNA levels of mutant KRAS as a sensitive marker of response to chemotherapy in pancreatic cancer. Sci. Rep..

[21] Kruger S., Heinemann V., Ross C., Diehl F., Nagel D., Ormanns S., Liebmann S., Prinz-Bravin I., Westphalen C.B., Haas M. (2018). Repeated mutKRAS ctDNA measurements represent a novel and promising tool for early response prediction and therapy monitoring in advanced pancreatic cancer. Ann. Oncol..

[22] Perets R., Greenberg O., Shentzer T., Semenisty V., Epelbaum R., Bick T., Sarji S., Ben-Izhak O., Sabo E., Hershkovitz D. (2018). Mutant KRAS Circulating Tumor DNA Is an Accurate Tool for Pancreatic Cancer Monitoring. Oncologist.

[23] Pietrasz D., Pécuchet N., Garlan F., Didelot A., Dubreuil O., Doat S., Imbert-Bismut F., Karoui M., Vaillant J.C., Taly V. (2017). Plasma Circulating Tumor DNA in Pancreatic Cancer Patients Is a Prognostic Marker. Clin. Cancer Res. Off. J. Am. Assoc. Cancer Res..

[24] Schlick K., Markus S., Huemer F., Ratzinger L., Zaborsky N., Clemens H., Neureiter D., Neumayer B., Beate A.-S., Florian S. (2021). Evaluation of circulating cell-free KRAS mutational status as a molecular monitoring tool in patients with pancreatic cancer. Pancreatology.

[25] Hindson B.J., Ness K.D., Masquelier D.A., Belgrader P., Heredia N.J., Makarewicz A.J., Bright I.J., Lucero M.Y., Hiddessen A.L., Legler T.C. (2011). High-throughput droplet digital PCR system for absolute quantitation of DNA copy number. Anal. Chem..

[26] Hindson C.M., Chevillet J.R., Briggs H.A., Gallichotte E.N., Ruf I.K., Hindson B.J., Vessella R.L., Tewari M. (2013). Absolute quantification by droplet digital PCR versus analog real-time PCR. Nat. Methods.

[27] Cohen J.D., Javed A.A., Thoburn C., Wong F., Tie J., Gibbs P., Schmidt C.M., Yip-Schneider M.T., Allen P.J., Schattner M. (2017). Combined circulating tumor DNA and protein biomarker-based liquid biopsy for the earlier detection of pancreatic cancers. Proc. Natl. Acad. Sci. USA.

[28] Honda K., Kobayashi M., Okusaka T., Rinaudo J.A., Huang Y., Marsh T., Sanada M., Sasajima Y., Nakamori S., Shimahara M. (2015). Plasma biomarker for detection of early stage pancreatic cancer and risk factors for pancreatic malignancy using antibodies for apolipoprotein-AII isoforms. Sci. Rep..

[29] Borrebaeck C.A.K. (2017). Precision diagnostics: Moving towards protein biomarker signatures of clinical utility in cancer. Nat. Rev. Cancer.

[30] Klett H., Fuellgraf H., Levit-Zerdoun E., Hussung S., Kowar S., Küsters S., Bronsert P., Werner M., Wittel U., Fritsch R. (2018). Identification and Validation of a Diagnostic and Prognostic Multi-Gene Biomarker Panel for Pancreatic Ductal Adenocarcinoma. Front. Genet..

[31] Hussung S., Follo M., Klar R.F.U., Michalczyk S., Fritsch K., Nollmann F., Hipp J., Duyster J., Scherer F., von Bubnoff N. (2020). Development and Clinical Validation of Discriminatory Multitarget Digital Droplet PCR Assays for the Detection of Hot Spot KRAS and NRAS Mutations in Cell-Free DNA. J. Mol. Diagn..

[32] Cox D.R. (1972). Regression Models and Life-Tables. J. R. Stat. Soc. Ser. B Methodol..

[33] Friedman J., Hastie T., Tibshirani R. (2010). Regularization Paths for Generalized Linear Models via Coordinate Descent. J. Stat. Softw..

[34] (2018). R.C.T. R: A Language and Environment for Statistical Computing. R Foundation for Statistical Computing, Vienna. https://www.R-project.org.

[35] Kim J.E., Lee K.T., Lee J.K., Paik S.W., Rhee J.C., Choi K.W. (2004). Clinical usefulness of carbohydrate antigen 19-9 as a screening test for pancreatic cancer in an asymptomatic population. J. Gastroenterol. Hepatol..

[36] Bernard V., Kim D.U., San Lucas F.A., Castillo J., Allenson K., Mulu F.C., Stephens B.M., Huang J., Semaan A., Guerrero P.A. (2019). Circulating Nucleic Acids Are Associated With Outcomes of Patients With Pancreatic Cancer. Gastroenterology.

[37] Sausen M., Phallen J., Adleff V., Jones S., Leary R.J., Barrett M.T., Anagnostou V., Parpart-Li S., Murphy D., Kay Li Q. (2015). Clinical implications of genomic alterations in the tumour and circulation of pancreatic cancer patients. Nat. Commun..

[38] Cheng H., Liu C., Jiang J., Luo G., Lu Y., Jin K., Guo M., Zhang Z., Xu J., Liu L. (2017). Analysis of ctDNA to predict prognosis and monitor treatment responses in metastatic pancreatic cancer patients. Int. J. Cancer.

[39] Patel H., Okamura R., Fanta P., Patel C., Lanman R.B., Raymond V.M., Kato S., Kurzrock R. (2019). Clinical correlates of blood-derived circulating tumor DNA in pancreatic cancer. J. Hematol. Oncol..

[40] Gall T.M.H., Belete S., Khanderia E., Frampton A.E., Jiao L.R. (2019). Circulating Tumor Cells and Cell-Free DNA in Pancreatic Ductal Adenocarcinoma. Am. J. Pathol..

[41] Hussung S., Akhoundova D., Hipp J., Follo M., Klar R.F.U., Philipp U., Scherer F., von Bubnoff N., Duyster J., Boerries M. (2021). Longitudinal analysis of cell-free mutated KRAS and CA 19–9 predicts survival following curative resection of pancreatic cancer. BMC Cancer.

[42] Conroy T., Hammel P., Hebbar M., Ben Abdelghani M., Wei A.C., Raoul J.L., Choné L., Francois E., Artru P., Biagi J.J. (2018). FOLFIRINOX or Gemcitabine as Adjuvant Therapy for Pancreatic Cancer. N. Engl. J. Med..

[43] Von Hoff D.D., Ervin T., Arena F.P., Chiorean E.G., Infante J., Moore M., Seay T., Tjulandin S.A., Ma W.W., Saleh M.N. (2013). Increased Survival in Pancreatic Cancer with nab-Paclitaxel plus Gemcitabine. N. Engl. J. Med..

[44] Golan T., Hammel P., Reni M., Van Cutsem E., Macarulla T., Hall M.J., Park J.-O., Hochhauser D., Arnold D., Oh D.-Y. (2019). Maintenance Olaparib for Germline BRCA-Mutated Metastatic Pancreatic Cancer. N. Engl. J. Med..

[45] Reiss K.A., Mick R., O’Hara M.H., Teitelbaum U., Karasic T.B., Schneider C., Cowden S., Southwell T., Romeo J., Izgur N. (2021). Phase II Study of Maintenance Rucaparib in Patients With Platinum-Sensitive Advanced Pancreatic Cancer and a Pathogenic Germline or Somatic Variant in BRCA1, BRCA2, or PALB2. J. Clin. Oncol. Off. J. Am. Soc. Clin. Oncol..

[46] Hegewisch-Becker S., Aldaoud A., Wolf T., Krammer-Steiner B., Linde H., Scheiner-Sparna R., Hamm D., Jänicke M., Marschner N. (2019). Results from the prospective German TPK clinical cohort study: Treatment algorithms and survival of 1,174 patients with locally advanced, inoperable, or metastatic pancreatic ductal adenocarcinoma. Int. J. Cancer.

[47] Kinugasa H., Nouso K., Miyahara K., Morimoto Y., Dohi C., Tsutsumi K., Kato H., Matsubara T., Okada H., Yamamoto K. (2015). Detection of K-ras gene mutation by liquid biopsy in patients with pancreatic cancer. Cancer.

[48] Castells A., Puig P., Móra J., Boadas J., Boix L., Urgell E., Solé M., Capellà G., Lluís F., Fernández-Cruz L. (1999). K-ras mutations in DNA extracted from the plasma of patients with pancreatic carcinoma: Diagnostic utility and prognostic significance. J. Clin. Oncol. Off. J. Am. Soc. Clin. Oncol..

[49] Chen H., Tu H., Meng Z.Q., Chen Z., Wang P., Liu L.M. (2010). K-ras mutational status predicts poor prognosis in unresectable pancreatic cancer. Eur. J. Surg. Oncol..

[50] Fleischhacker M., Schmidt B. (2007). Circulating nucleic acids (CNAs) and cancer—A survey. Biochim. Et Biophys. Acta.

[51] Bronkhorst A.J., Ungerer V., Holdenrieder S. (2019). The emerging role of cell-free DNA as a molecular marker for cancer management. Biomol. Detect. Quantif..

[52] Marchese R., Muleti A., Pasqualetti P., Bucci B., Stigliano A., Brunetti E., De Angelis M., Mazzoni G., Tocchi A., Brozzetti S. (2006). Low correspondence between K-ras mutations in pancreatic cancer tissue and detection of K-ras mutations in circulating DNA. Pancreas.

