# Supplementary material for: Integrated Analysis of Cell-Free DNA and Novel Protein Biomarkers for Stratification and Therapy Monitoring in Stage IV Pancreatic Cancer: A Preliminary Study
Source: Diagnostics (Basel). 2024 Dec 28;15(1):49. doi: 10.3390/diagnostics15010049 (PMC11720586; doi:10.3390/diagnostics15010049)
Supplement: Supplementary file 1 [file diagnostics-15-00049-s001.zip › diagnostics-3374351-supplementary.pdf]

diagnostics

MDPI

Supplementary Materials:

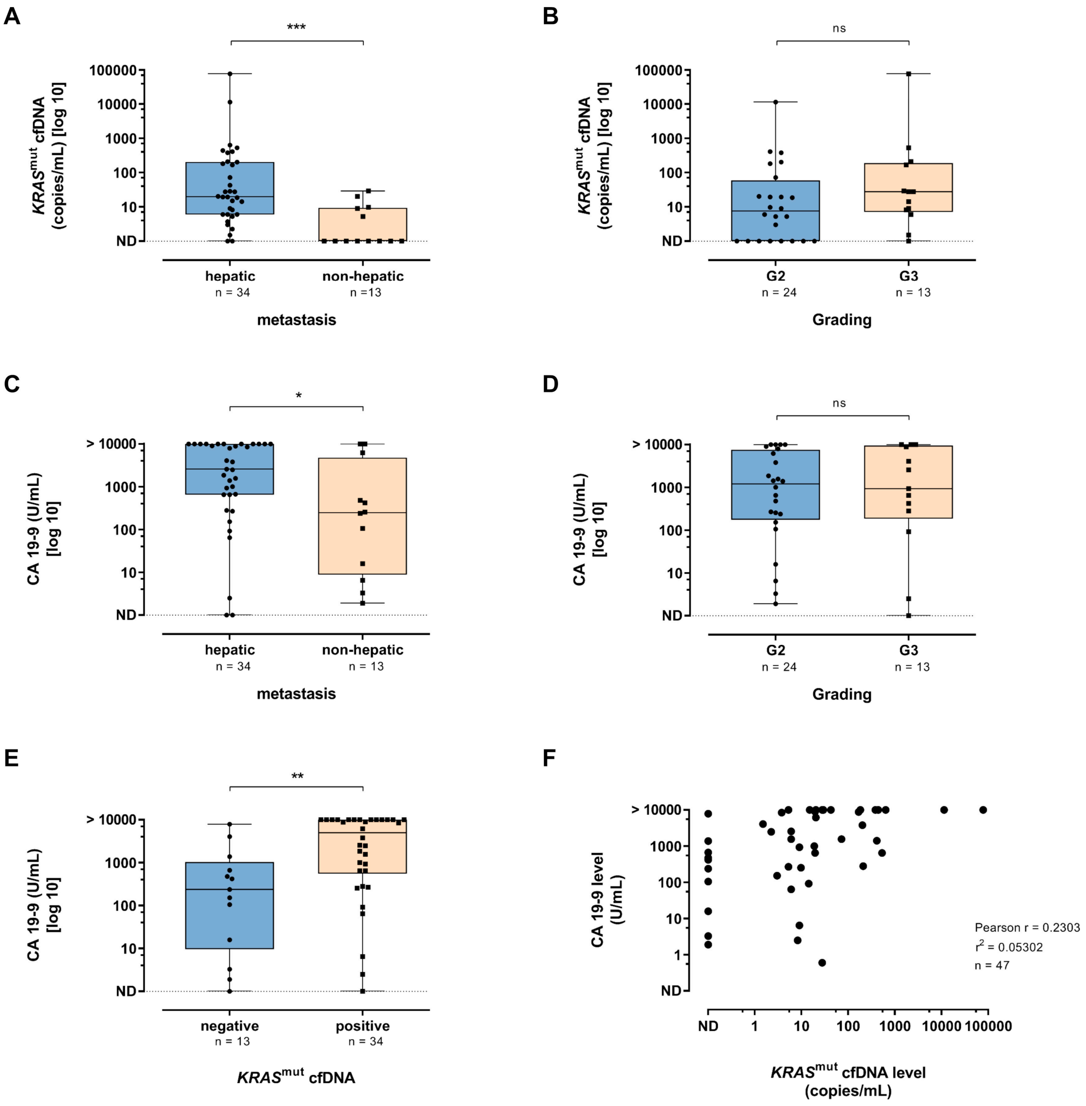

Supplemental Figure S1. Plasma *KRAS*^{mut} cfDNA and CA 19-9 in metastasized PDAC patients (A) Plasma *KRAS*^{mut} cfDNA level in untreated metastasized PDAC patients was compared to hepatic and non-hepatic metastasis. (B) *KRAS*^{mut} cfDNA copies/ml were analyzed and compared to tumor grading. (C) CA 19-9 level in untreated metastasized PDAC patients was compared to hepatic and non-hepatic metastasis. (D) CA 19-9 U/ml were analyzed and compared to tumor grading. (E) CA 19-9 level was compared to *KRAS* positive and negative untreated metastasized PDAC patients. (F) CA 19-9 level was compared to *KRAS*^{mut} cfDNA level in untreated metastasized PDAC patients. PDAC, pancreatic ductal adenocarcinoma.

 https://doi.org/10.3390/diagnostics15010049

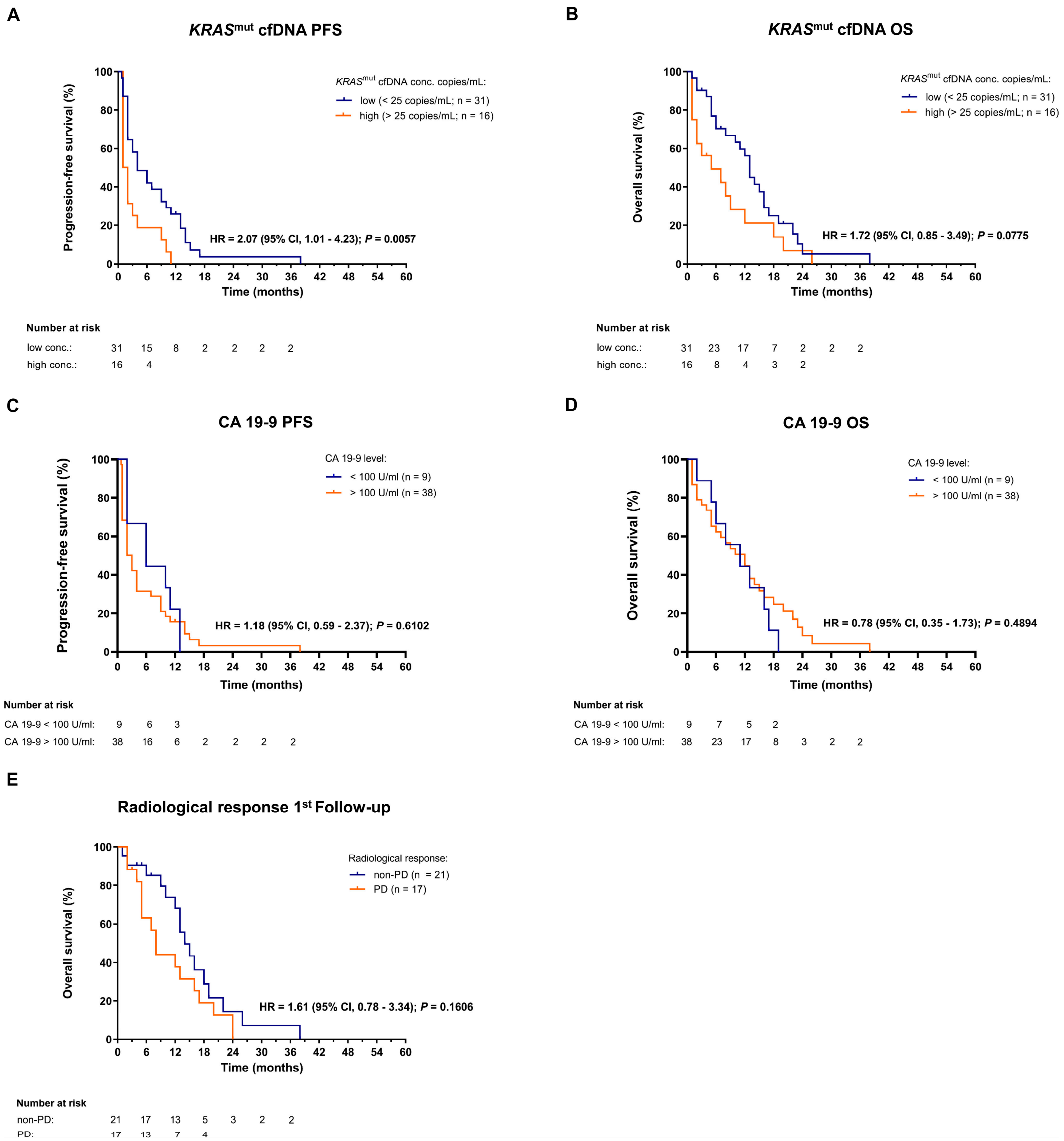

Supplemental Figure S2. Association of *KRAS*mut cfDNA and CA 19-9 detection with survival endpoints
(A, B) Kaplan-Meier estimates of PFS (A) and OS (B) for metastatic PDAC patients with versus without high *KRAS*mut cfDNA levels (> 25 copies/mL) before the start of first-line systemic treatment. (C, D) Kaplan-Meier estimates of PFS (C) and OS (D) for metastatic PDAC patients with versus without elevated CA 19-9 levels before start of first-line systemic treatment. (E) Kaplan-Meier estimate of OS for metastasized PDAC patients stratified by radiological response at time of first restaging: non-PD versus PD. OS, overall survival; PD, progressive disease; PDAC, pancreatic ductal adenocarcinoma; PFS, progression-free survival; PR, partial response; SD, stable disease. *P < 0,05, **P < 0,01 und ***P < 0,001.

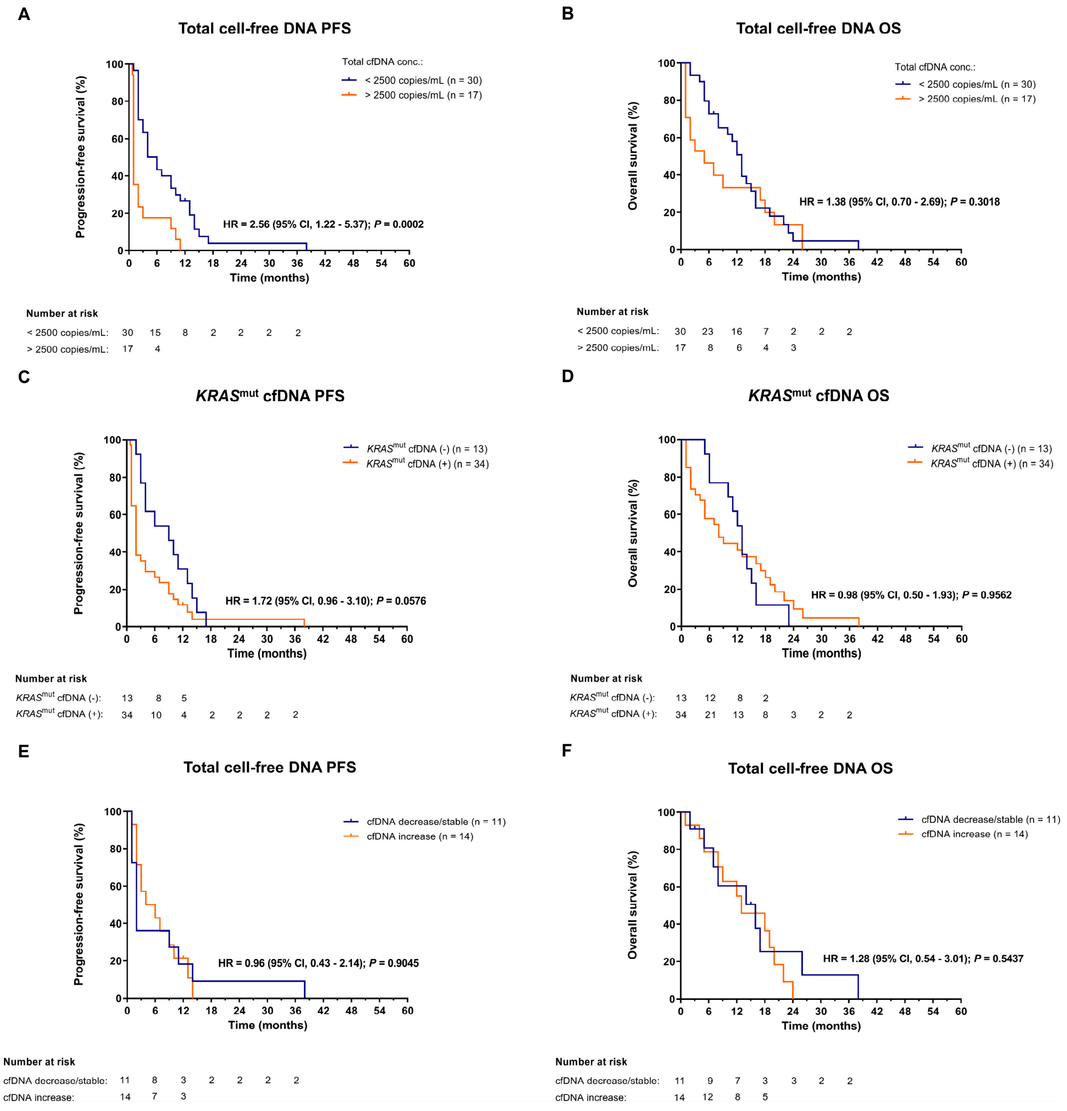

Supplemental Figure S3. Association of *KRAS*^{mut} cfDNA detection with survival endpoints (A, B) Kaplan-Meier estimates of PFS (A) and OS (B) for metastatic PDAC patients with versus without detectable total cell-free DNA levels over 2500 copies/mL before the start of palliative treatment. (C, D) Kaplan-Meier estimates of PFS (D) and OS (D) for metastatic PDAC patients with versus without detectable *KRAS*^{mut} cfDNA in plasma before the start of palliative treatment. (E, F) Kaplan-Meier estimates of progression-free survival (E) and overall survival (F) for metastasized PDAC patients stratified by total cell-free DNA change at time of first restaging: total cell-free DNA decrease versus increase. OS, overall survival; PDAC, pancreatic ductal adenocarcinoma; PFS, progression-free survival.

Supplemental Table S1. Basic clinical characteristics of the study cohort

	**Number of patients**	**Percentage (%)**

**1st line palliative therapy**		
**Gemcitabine monotherapy**	6	12.8
**Gemcitabine+nab-Paclitaxel**	9	19.1
**FOLFIRINOX**	26	55.3
**FOLFOX**	1	2.1
**Best Supportive Care (BSC)**	5	10.6
***KRAS*mut cfDNA (plasma)**		
**Wildtype**	8	17.0
**c.35G>T-p.G12V (Valin)**	12	25.5
**c.35G>A-p.G12D (Aspartat)**	17	36.2
**c.34G>C-p.G12R (Arginin)**	5	10.6
**c.34G>T-p.G12C (Cystein)**	2	4.3
**c.183A>C-p.Q61H (Histidin)**	1	2.1
**c.182A>G-p.Q61R (Arginin)**	2	4.3
	**Median**	**Range/95% CI**
**Age (years)**	67	42 - 84
**Time to progression (months)**	3.0	2 - 6
**Overall survival (months)**	9.0	5 - 13

CI, Confidence interval

Supplemental Table S2. Clinical characteristics of the individual patients

ID	Gender	Age	Tumor location	UICC Stage	Site of Metastasis	Survival from diagnosis (months)	*KRAS* mutation	Regimen 1st line
#000007	m	72	Pancreas body	IV	HEP	1.0	c.35G>A	Gemcitabine
#000051	w	79	Pancreas body	IV	PER	8.0	c.35G>A	Gemcitabine
#000048	w	46	Pancreas tail	IV	HEP	19.0	c.35G>T	FOLFIRINOX

#000055	m	79	Pancreas head	IV	OTH	13.0	Not detected	Best supportive care
#000056	w	51	Overlap	IV	HEP, PER, LYM	1.0	c.35G>T	FOLFOX
#000070	w	52	Pancreas head	IV	HEP	3.0	c.35G>A	FOLFIRINOX
#000075	w	76	Pancreas head	IV	PER	11.0	Not detected	Gemcitabine
#000076	m	67	Pancreas tail	IV	HEP	18.0	c.35G>T	FOLFIRINOX
#000081	w	74	Pancreas head	IV	HEP	1.0	c.35G>A	Gemcitabine
#000088	w	79	Pancreas head	IV	HEP	6.0	c.35G>T	Gemcitabine/nab-Paclitaxel
#000104	m	60	Pancreas tail	IV	HEP	22.0	c.35G>A	FOLFIRINOX
#000113	w	67	Pancreas body	IV	PER	38.0	c.35G>T	Gemcitabine/nab-Paclitaxel
#000116	w	65	Pancreas tail	IV	HEP, LYM	7.0	c.35G>A	FOLFIRINOX
#000123	w	77	Overlap	IV	HEP	5.0	c.35G>T	Gemcitabine
#000132	m	78	Pancreas head	IV	HEP	5.0	c.35G>T	Gemcitabine
#000146	w	76	Pancreas body	IV	HEP, PER, PUL	8.0	c.35G>T	Gemcitabine/nab-Paclitaxel
#000157	m	82	Overlap	IV	PER	6.0	Not detected	Best supportive care
#000169	w	42	Pancreas body	IV	HEP, LYM, PUL	13.0	c.35G>A	FOLFIRINOX
#000172	m	78	Pancreas head	IV	HEP	2.0	c.35G>A	Best supportive care
#000189	m	81	Pancreas body	IV	HEP, PUL	12.0	c.35G>T	Gemcitabine/nab-Paclitaxel
#000192	w	70	Pancreas head	IV	PER, LYM, OSS, OTH	2.0	c.35G>A	FOLFIRINOX
#000221	w	55	Pancreas head	IV	HEP	24.0	c.35G>A	FOLFIRINOX
#000255	m	58	Pancreas tail	IV	HEP, PER, OSS, OTH	12.0	c.34G>C	FOLFIRINOX
#000281	m	84	Pancreas head	IV	HEP	2.0	c.35G>T	Best supportive care
#000324	m	77	Pancreas body	IV	HEP, PER, LYM	20.0	c.35G>A	Gemcitabine/nab-Paclitaxel
#000305	w	66	Pancreas body	IV	PER	15.0	Not detected	FOLFIRINOX
#000326	m	75	Pancreas head	IV	PER	15.0	c.183A>C	Gemcitabine/nab-Paclitaxel
#000345	m	61	Pancreas head	IV	HEP	3.0	c.35G>A	FOLFIRINOX
#000337	m	72	Pancreas body	IV	HEP	17.0	c.34G>T	FOLFIRINOX
#000356	w	52	Pancreas body	IV	HEP, PER	1.0	c.34G>C	FOLFIRINOX

#000378	m	54	Pancreas tail	IV	PER	14.0	c.35G>T& G>A	FOLFIRINOX
#000379	m	66	Pancreas tail	IV	HEP, PUL	12.0	c.35G>T	FOLFIRINOX
#000380	w	61	Overlap	IV	PER	16.0	Not detected	FOLFIRINOX
#000407	m	67	Pancreas head	IV	HEP	10.0	Not detected	FOLFIRINOX
#000409	w	48	Overlap	IV	HEP	26.0	c.35G>A	FOLFIRINOX
#000435	m	55	Pancreas head	IV	HEP	5.0	c.35G>A	FOLFIRINOX
#000395	w	62	Overlap	IV	PUL	20.0	c.34G>C	Gemcitabine/nab-Paclitaxel
#000413	m	74	Pancreas tail	IV	HEP	16.0	c.182A>G	Gemcitabine/nab-Paclitaxel
#000383	w	69	Pancreas body	IV	HEP, PUL	7.0	c.35G>A	FOLFIRINOX
#000447	m	53	Overlap	IV	HEP	9.0	c.35G>A	FOLFIRINOX
#000484	m	66	Pancreas head	IV	PER	2.0	c.34G>C	Gemcitabine/nab-Paclitaxel
#000491	w	48	Pancreas body	IV	HEP	4.0	c.182A>G	FOLFIRINOX
#000507	w	70	Pancreas head	IV	PUL	23.0	Not detected	FOLFIRINOX
#000520	m	69	Pancreas body	IV	HEP	4.0	c.34G>T	FOLFIRINOX
#000523	w	63	Pancreas head	IV	PER	5.0	Not detected	FOLFIRINOX
#000536	m	52	Pancreas head	IV	HEP, LYM	13.0	c.35G>A	FOLFIRINOX
#000589	w	81	Pancreas tail	IV	HEP, PER, LYM, PUL, OTH	1.0	c.34G>C	Best supportive care

Supplemental Table S3. *KRAS*^{mut} cfDNA status and radiological site of metastasis

		SITE OF METASTASIS					
**PATIENT**	**ctDNA**	**HEP**	**PER**	**LYM**	**PUL**	**OSS**	**OTH**
**#000048**	**+**	X					
**#000070**	**+**	X					

**#000076**	+	X					
**#000081**	+	X					
**#000104**	+	X					
**#000123**	+	X					
**#000132**	+	X					
**#000172**	+	X					
**#000221**	+	X					
**#000281**	+	X					
**#000345**	+	X					
**#000337**	+	X					
**#000409**	+	X					
**#000435**	+	X					
**#000413**	+	X					
**#000447**	+	X					
**#000491**	+	X					
**#000520**	+	X					
**#000007**	+	X		X			
**#000116**	+	X		X			
**#000056**	+	X	X	X			
**#000324**	+	X	X	X			
**#000356**	+	X	X				
**#000146**	+	X	X		X		
**#000379**	+	X			X		
**#000383**	+	X			X		
**#000589**	+	X	X	X	X		X
**#000255**	+	X	X			X	X
**#000192**	+		X	X		X	X
**#000051**	+		X				
**#000113**	+		X				
**#000484**	+		X				
**#000395**	+				X		
**#000507**	-				X		
**#000189**	-	X			X		
**#000169**	-	X		X	X		
**#000536**	-	X		X			
**#000088**	-	X					
**#000407**	-	X					
**#000523**	-		X				
**#000075**	-		X				
**#000157**	-		X				
**#000305**	-		X				
**#000326**	-		X				

**#000378**	-		X				
**#000380**	-		X				
**#000055**	-						X

ctDNA, circulating tumor DNA; HEP, liver; PER, peritoneum; LYM, lymph node; PUL, lung; OSS, bones; OTH, other

Supplemental Table S4. Progression-free survival analysis by clinico-pathologic variables and *KRAS*^{mut} cfDNA status

Variable	Univariate analysis			Multivariate analysis		
	HR	95% CI§	*P*	HR	95% CI	*P*
Age						
≥ median vs < median	1.15	0.645-2.049	0.6256	4.39	1.250-15.42	**0.021**
Gender						
male vs female	1.14	0.639-2.044	0.6370			
Tumor location pancreas						
body & tail vs head	0.75	0.404-1.403	0.3463	0.20	0.053-0.721	**0.014**
Tumor differentiation						
poor vs medium/well	1.46	0.733-2.905	0.2382			
Liver metastasis						
present vs absent	2.33	1.304-4.146	**0.0032**			
No. of metastatic site						
≥2 vs 1	1.95	0.923-4.108	**0.0274**	7.20	1.149-45.080	**0.035**
Palliative chemotherapy						
yes vs no	0.75	0.262-2.121	0.5277			
CA 19-9 status						
> 37 vs ≤ 37 U/mL	1.23	0.552-2.730	0.6314	20.6	1.382-305.76	**0.028**
KRAS^{mut} cfDNA status						
positive vs negative	2.19	1.226-3.896	**0.0093**			
CA 19-9 during follow-up						
increase vs decrease	1.25	0.533-2.937	0.5871			

KRAS^{mut} during follow-up						
increase vs decrease	3.98	1.009-15.70	**0.0003**	10.9	2.575-46.444	**0.001**

§ CI, confidence interval

Supplemental Table S5. Prognostic impact of the single proteins by considering the impact of therapy

Protein	**HR$**	**95% CI§**	**P Value**
CEMIP	2.04	0.69 – 6.05	0.21
COL10A1	0.90	0.29 – 2.75	0.85
HGF	1.85	0.61 – 5.58	0.27
LAMB3	3.42	0.99 – 11.60	0.044 *
POSTN	1.58	0.52 – 4.82	0.42
SERPINB5	3.50	1.02 – 12.00	0.037 *
SFN	1.65	0.54 – 5.07	0.37
SPP1	2.94	0.90 – 9.57	0.071
TFF1	1.60	0.54 – 4.77	0.39
TMPRSS4	1.19	0.41 – 3.49	0.75
KRAS^{mut} cfDNA	7.36	1.79 – 30.3	0.0042*

$HR, hazard ratio, § CI, confidence interval, * values < 0.05 are considered significant

Supplemental Table S6. Significance of the selected biomarkers in the multivariate CoxPH model

Feature	**Coefficient**	**P Value**
CEMIP	0.8447	0.0543 *
KRAS^{mut} cfDNA	0.7393	0.1019

TFF1	0.5526	0.1046
LAMB3	0.4119	0.4330
HGF	0.3503	0.5192
TMPRSS4	0.2986	0.3572
SERPINB5	0.2589	0.5480

* values < 0.1 are considered significant

Supplemental Table S7. Significance of the selected biomarkers in the risk classifier

Feature	**Coefficient**
CEMIP	0.0000
KRAS^{mut} cfDNA	0.0861
TFF1	0.0349
LAMB3	-0.0171
HGF	-10.4303
TMPRSS4	0.0000
SERPINB5	0.0860

Supplemental Table S8. Risk labels provided by CoxPH and by the risk classifier

**Sample**	**Group**	**Risk group provided by CoxPH**	**Predicted risk group by risk classifier**
#000048	PT-and-UT	low	low
#000051	**PT-and-UT**	**high**	**low**
#000088	PT-and-UT	high	high
#000104	**PT-and-UT**	**low**	**low**
#000113	PT-and-UT	low	high
#000116	**PT-and-UT**	**high**	**high**
#000123	PT-and-UT	high	high
#000132	**PT-and-UT**	**high**	**high**
#000146	PT-and-UT	high	high

#000305	**PT-and-UT**	**low**	**low**
#000326	PT-and-UT	low	low
#000324	**PT-and-UT**	**low**	**low**
#000345	PT-and-UT	low	low
#000379	**PT-and-UT**	**low**	**high**
#000380	PT-and-UT	high	high
#000407	**PT-and-UT**	**high**	**high**
#000447	PT-and-UT	high	high
#000520	**PT-and-UT**	**high**	**high**
#000523	PT-and-UT	high	high
#000055	**PT-only**	**-**	**high**
#000056	PT-only	-	low
#000070	**PT-only**	**-**	**high**
#000075	PT-only	-	low
#000157	**PT-only**	**-**	**high**
#000172	PT-only	-	high
#000192	**PT-only**	**-**	**high**
#000281	PT-only	-	high
#000356	**PT-only**	**-**	**high**
#000395	PT-only	-	high
#000409	**PT-only**	**-**	**low**
#000435	PT-only	-	high
#000484	**PT-only**	**-**	**high**
#000491	PT-only	-	high
#000507	**PT-only**	**-**	**low**
#000536	PT-only	-	high
#000589	**PT-only**	**-**	**high**
